# Time-resolved connectome of the five-factor model of personality

**DOI:** 10.1038/s41598-019-51469-2

**Published:** 2019-10-21

**Authors:** L. Passamonti, R. Riccelli, I. Indovina, A. Duggento, A. Terracciano, N. Toschi

**Affiliations:** 1 Institute of Bioimaging & Molecular Physiology, National Research Council, Milano, Italy; 2 Department of Clinical Neurosciences, University of Cambridge, Cambridge, UK; 3 Laboratory of Neuromotor Physiology, IRCCS Santa Lucia Foundation, 00179 Rome, Italy; 4 Saint Camillus International University of Health and Medical Sciences, 00131 Rome, Italy; 5 Department of Biomedicine & Prevention, University “Tor Vergata”, Rome, Italy; 6 Department of Geriatrics, Florida State University College of Medicine, Tallahassee, USA; 7 Department of Radiology, Martinos Center for Biomedical Imaging, Boston & Harvard medical School, Boston, USA

**Keywords:** Personality, Statistics

## Abstract

The human brain is characterized by highly dynamic patterns of functional connectivity. However, it is unknown whether this time-variant ‘connectome’ is related to the individual differences in the behavioural and cognitive traits described in the five-factor model of personality. To answer this question, inter-network time-variant connectivity was computed in *n* = 818 healthy people via a dynamical conditional correlation model. Next, network dynamicity was quantified throughout an *ad-hoc* measure (T-index) and the generalizability of the multi-variate associations between personality traits and network dynamicity was assessed using a train/test split approach. Conscientiousness, reflecting enhanced cognitive and emotional control, was the sole trait linked to stationary connectivity across several circuits such as the default mode and prefronto-parietal network. The stationarity in the ‘communication’ across large-scale networks offers a mechanistic description of the capacity of conscientious people to ‘protect’ non-immediate goals against interference over-time. This study informs future research aiming at developing more realistic models of the brain dynamics mediating personality differences.

## Introduction

Understanding *how* people differ in their cognitive, emotional, and behavioural dispositions is a central theme in psychology, psychiatry, and neurology. At the intersection across these disciplines, the ‘personality neuroscience’ field has emerged as a new academic ‘arena’ where researchers strive to reveal the brain mechanisms of personality traits^1,2^. So far, several neuroimaging studies have focused on linking personality measures with diverse structural and functional measures in single brain regions^1,3–19^. Others have investigated how variability in time-averaged (or static) functional connectivity relates to personality^7,18–25^.

However, no-one has yet assessed how the dynamic patterns of ‘communications’ across large-scale brain networks mediate personality differences. To offer new insights into the core neurological underpinnings of personality, we need to transition from static measures of connectivity to indices that resolve the temporal component of such connectivity patterns^26,27^. The most appropriate method to reveal the dynamicity (or its reverse, stationarity) in the connectivity between two ‘nodes’ (in this and other studies ‘a node’ is a ‘large-scale’ network) has been the subject of a fruitful debate^28^. Although different definitions exist^28^, a non-stationary connection can be quantified by the presence of frequent, long-lasting, or high excursions in the connection strength, despite the average value of the connection strength *itself* and the directionality of the excursion. In other words, a ‘connection’ between two ‘nodes’ (i.e., the amplitude of the correlation between two time-series) is ‘dynamic’ when it fluctuates frequently, for a long time, or to a large extent (or any combination of these elements). In contrast, a ‘stationary’ connection is one in which these connectivity patterns are minimal or infrequent.

At the cognitive level, the degree of dynamicity or stationarity in the human ‘connectome’ can be interpreted in different ways, depending on which ‘nodes’ are involved. For example, dynamic connections between networks that ‘feed’ external stimuli into the brain might result in ‘distractibility’, as, in this scenario, the rapidly changing information can ‘take the lead’ in driving the brain connections. Conversely, stationary connections between ‘top-down’ networks and ‘bottom-up’ sensory circuits can result in less ‘distractibility’, as, in this case, the incoming inputs may be ‘stabilized’ over-time against interference.

 So far, different but partially related methods have been used to quantify the time-varying connectivity in the human brain, the most common being the ‘sliding’ window approach^27–31^. However, this method poses some limits on the temporal resolution and thus on the ability to characterize the relatively abrupt changes in the functional connectivity patterns that are commonly observed in resting-state functional magnetic resonance imaging (rs-fMRI) data^29^. Here, we employed a dynamic conditional correlation (DCC) model that has been specifically developed for rs-fMRI datasets^32^. The DCC is based on a point-by-point volatility model (a generalized autoregressive conditional heteroscedastic – GARCH -model) and is robust against apparent changes in correlations caused by random noise^30^. These features make it an attractive tool for resolving the time-variant associations in datasets with low signal-to-noise ratio such as the rs-fMRI data.

We also sought to identify the time-resolved functional connectivity patterns that related to the cognitive and behavioural dispositions described in the five-factor model (FFM) of personality^33,34^. The FFM traits (i.e., neuroticism, extraversion, openness, agreeableness, and conscientiousness) have been empirically derived from a large body of epidemiological and psychosocial research^35–38^. The FFM traits also display sufficient universality across different demographic and cultural groups and predict important outcome measures such as educational or occupational success, risk to develop dementia, and longevity^35–38^.

Overall, this study was exploratory in its nature as there was (to the best of our knowledge) no other study assessing the relationship between time-variant connectivity measures and personality traits. However, we had some expectations regarding a specific effect of conscientiousness on such time-resolved connectivity indices. This is because we found, in the same sample of participants used here, that static (i.e., time-averaged) ‘connectomic’ measures (i.e., nodal strength, local clustering, and betweenness-centrality) *positively* related to conscientiousness, but not to any other FFM trait^25^. Hence, here, we tested whether conscientiousness was the sole FFM trait linked to time-resolved connectivity measures, over and above its effect on ‘static’ connectivity indices.

Nevertheless, it was reasonable to hypothesize that other FFM traits could have been linked to more or less dynamic connectivity patterns (e.g., openness could have been associated to more dynamic connectivity across sensory-related circuits).

## Results

### Participants

All data were drawn from *n* = 818 individuals from the Human Connectome Project (HCP) database, a large repository of behavioural and neuroimaging measures. The demographic and personality variables of the HCP sample are summarized in Table 1. Most participants were right-handed white Americans. Less than 10% had a Hispanic or Latino background.

**Table 1:** Age, education, and personality data are expressed as mean ± standard deviation while the range in square brackets [] is expressed as minimum-maximum. NEO five-factors inventory questionnaire, NEO-FFI.

**Demographic variables**		
Gender (males/females)	367/451	
Age (years)	28.7 ± 3.7 [22–37]	
Handedness (Right/Left/Both)	743/73/2	
Education (years)	14.9 ± 1.8 [11–17]	
Ethnicity (%)	Hispanic/Latino	8.6%
	Not Hispanic/Latino	90.5%
	Unknown/Not Reported	0.9%
**Personality scores (NEO-FFI)**		
Neuroticism	16.3 ± 7.2 [0–43]	
Extraversion	30.7 ± 5.9 [11–47]	
Openness	28.3 ± 6.1 [12–45]	
Agreeableness	32.0 ± 5.0 [13–45]	
Conscientiousness	34.5 ± 5.9 [12–48]	

Potentially confounding variables such as age, sex, years of education, handedness, and intelligence scores were included as ‘nuisance’ variables in the statistical models testing for the relationship between the FFM personality traits and the time-resolved connectivity patterns.

### Neuroimaging findings

#### Independent components analysis (ICA) of large-scale networks ‘nodes’

**Figure 1:**
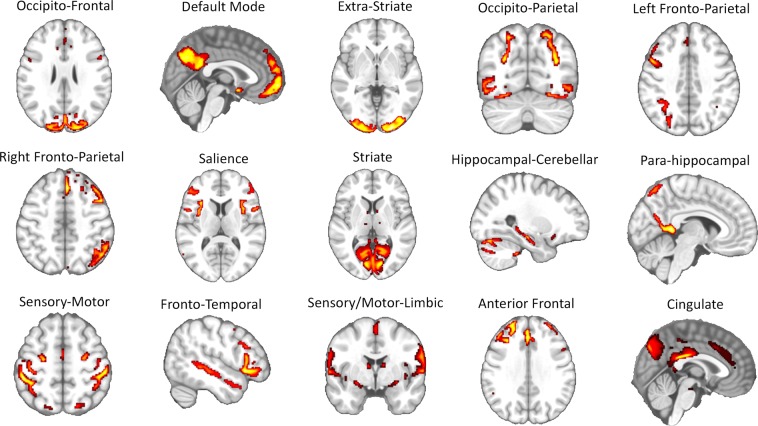
Resting-state ‘large-scale’ networks identified via independent component analysis (ICA). By using group ICA, the Human Connectome Project consortium has identified fifteen distinct brain circuits that we used to study the time-resolved connectivity patterns. The complete list of the brain region in each resting-state network is reported in Supplementary Table 1.

Brain networks were identified via group-ICA (dimensionality: *n* = 15) calculated by the HCP consortium and were characterized by a series of brain regions that have been reported in earlier studies (e.g., the sensory-motor circuit, the visual circuits, the default-mode network, the left and right fronto-parietal circuits, the salience network, etc.)^39,40^ (Fig. 1 and Supplementary Table 1 for a list of the anatomical regions belonging to each network ‘node’). Average connectivity strengths between nodes were also provided by the HCP consortium (‘netmats2’). Each of these large-scale networks was considered as a ‘node’ in the time-resolved connectivity analyses described in the Methods section.

#### Dynamical connectivity results independently of personality differences

**Figure 2:**
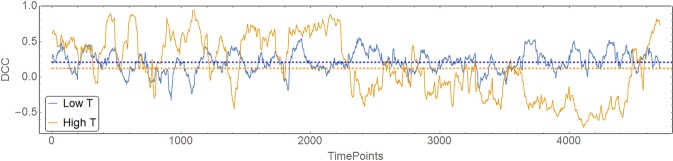
Examples of high- and low- dynamicity (i.e. T-index) in time-resolved connectivity. Two types of time-resolved connections are depicted, one with high-dynamicity (high “T”-index, light brown line) and the second with low-level of dynamicity (i.e. high stationarity) (low “T”-index, blue line). The dotted lines represent the median values for each connection (i.e. static/time-averaged connectivity). The more dynamic connection exhibits more, higher, and longer-lasting excursions from the overall median. DCC, dynamic conditional correlation.

First, the time-variant connectivity was estimated via the dynamic conditional correlation (DCC) model developed for rs-fMRI datasets^32^. A connection-wise index of non-stationarity (“T”-index) (initially introduced by Zalesky and colleagues) was computed for each connection (Fig. 2 for examples from real data)^27^. The higher the “T”-index, the more ‘dynamic’ (i.e., ‘non-stationary’) a connection is (see Methods section).

**Figure 3:**
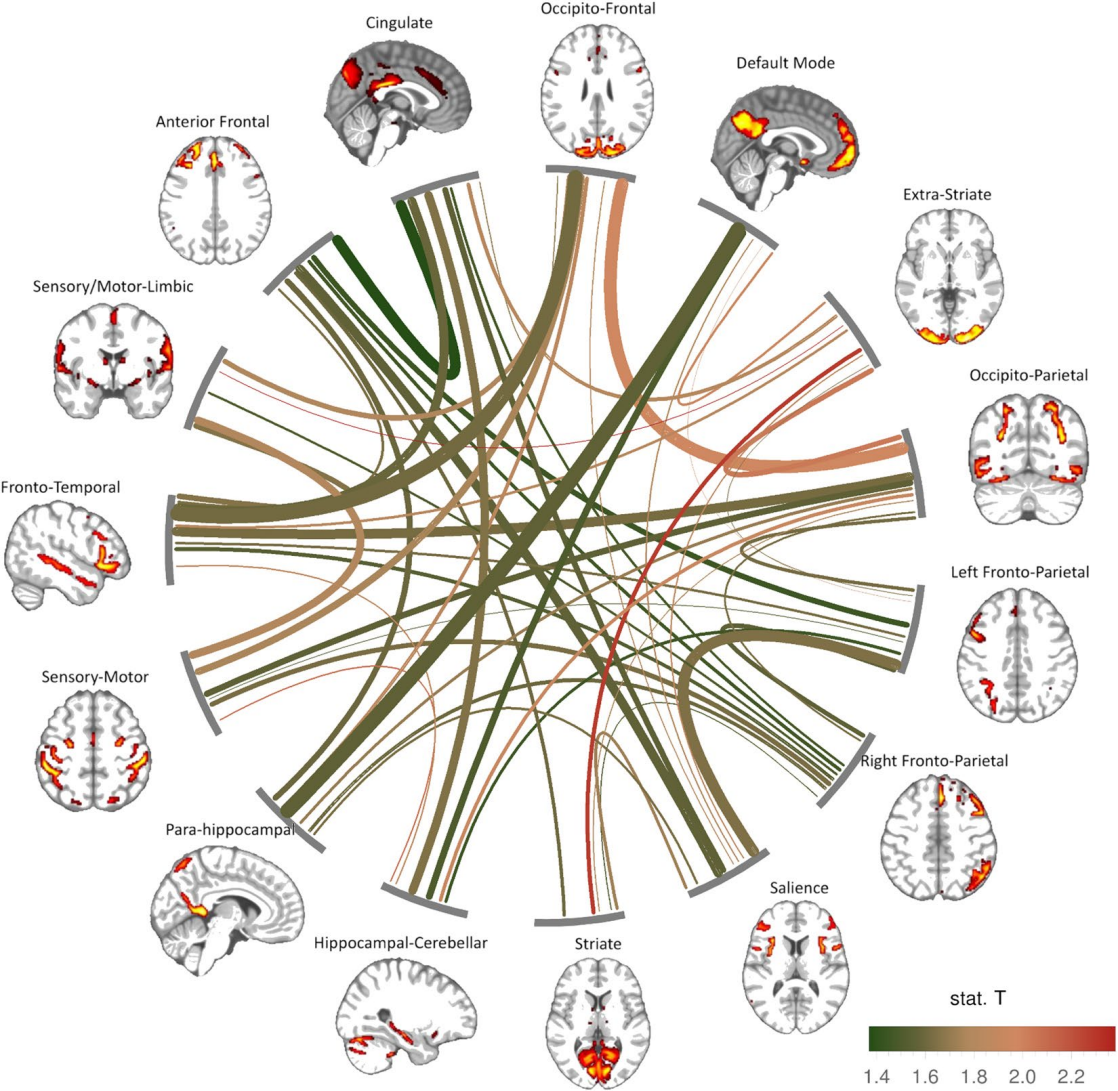
Dynamicity and strength of inter-network connectivity regardless of personality differences. The colour of the lines connecting each pair of ‘nodes’ (large scale-networks) reflects the degree of dynamicity (red: more dynamic, green: less dynamic, as assessed by the “T”-index, see Methods and Fig. 2). The thickness of each line represents the strength of the connection between each couple of nodes (with thicker lines reflecting stronger connections, as assessed by the HCP consortium in the ‘netmats2’ version). Both strength and dynamicity are represented through median values across all subjects. Only connections associated with positive strength (i.e. partial correlation, as provided by the HCP consortium) are shown.

Second, the dynamicity of each connection (i.e. its “T”-index) was plotted against the static strength of the same connection (obtained from the HCP consortium and calculated using partial correlation) as shown in Fig. 3.

Independently of personality differences, the connections between visuo-parietal networks (occipito-parietal/occipito-frontal) and visual circuits (striate/extrastriate) that ‘feed’ external input into the brain were amongst the most dynamic (Fig. 3). Connections between occipito-frontal and sensory/motor networks and within sensory-motor circuits (i.e., sensory/motor-limbic-sensory/motor) also displayed a relatively high level of dynamicity (Fig. 3). In contrast, the connections across networks involved in cognitive controls (e.g., cingulate network, default mode network, hippocampal/para-hippocampal circuits) showed more stationary patterns of connectivity (Fig. 3**)**.

#### Correlations between time-resolved indices of functional connectivity and personality traits

Next, we studied how the dynamicity of the connections across the nodes was related to individual differences in the FFM traits. To evaluate the generalizability of our findings, the initial sample of *n* = 818 participants was split into two sub-samples: a ‘training’ set (75% of participants, *n* = 613) and a ‘test’ set (25% of participants, *n* = 205). In the training set, we used multi-variate regression analyses (i.e., general linear models, GLMs) with the “T”-index as dependent variable, to explore the associations between the dynamicity of each connection and each FFM trait while accounting for potentially confounding effects driven by the remaining FFM traits as well as other ‘nuisance’ factors such as sex, age, education, handedness, and intelligence scores. Associations surviving a threshold of P < 0.05 (false-discovery-rate-FDR-corrected across 105 connections) were considered statistically significant. The associations discovered (i.e., the regression models estimated) in the ‘training’ dataset were used to assess the generalizability of our findings in the ‘test’ dataset (see **Methods**) using the relative root mean square error (RRMSE) as a criterion of merit^41^.

**Figure 4:**
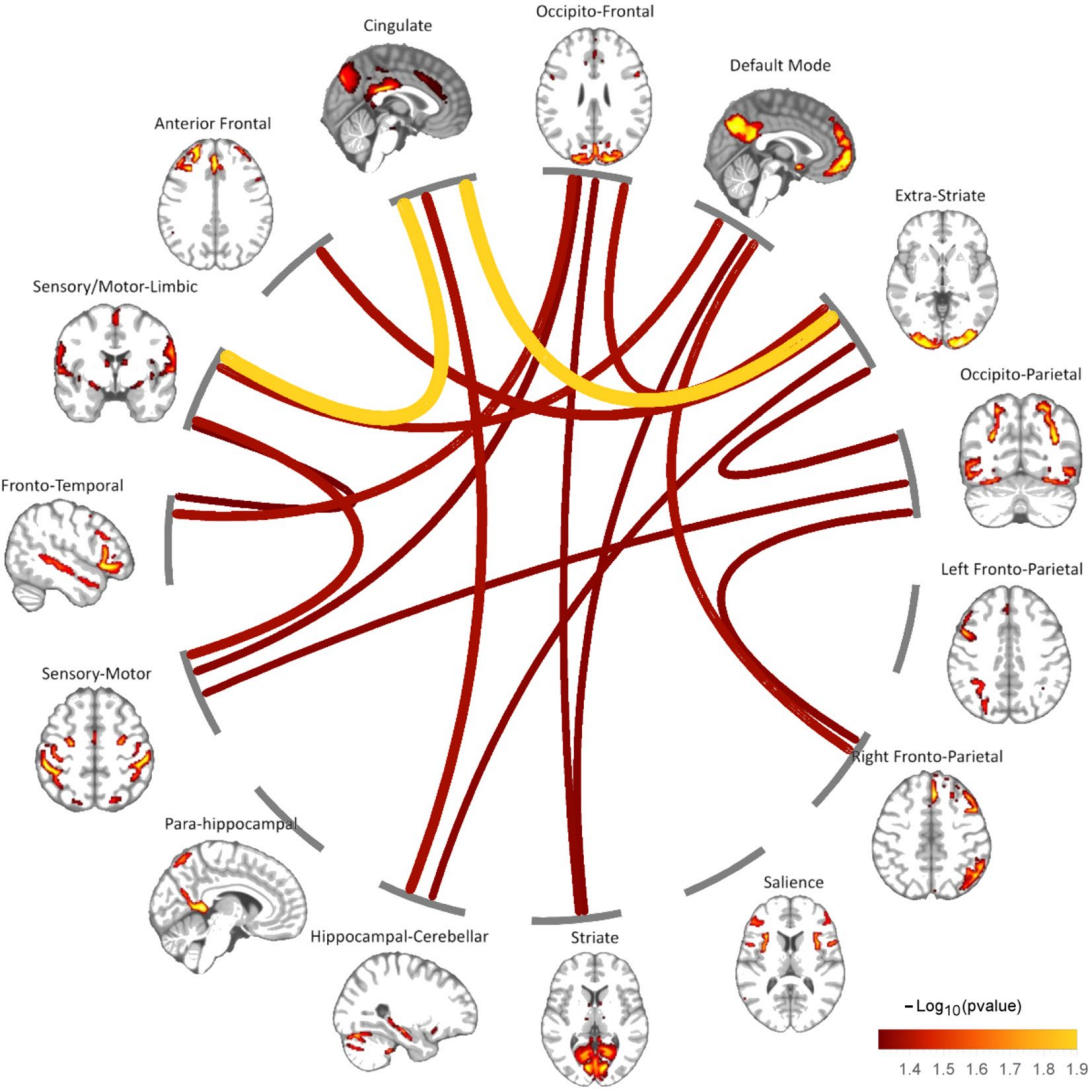
Conscientiousness was the only personality trait that was linked to stationary connectivity patterns across large-scale networks after accounting for potentially confounding factors such as the remaining personality traits, age, sex, years of education, handedness, and intelligence. The colour of the lines connecting each pair of ‘nodes’ (large scale-networks) represents the statistical significance (−Log_10_−p-value, yellow colour representing lower p-values: e.g. −Log_10_(0.05) = 1.30 and −Log_10_(0.01) = 2) of the negative associations found between conscientiousness scores and the “T”-index of each connection. The thickness of each line represents the effect sizes for each connection with thicker lines reflecting higher effect sizes. All associations shown survive a threshold of P < 0.05, correction for multiple comparisons using false discovery rate across all possible 105 connections (see Table 2).

No significant associations, either positive or negative, were found between neuroticism (P’s > 0.2), extraversion (P’s > 0.6), openness (P’s > 0.2), and agreeableness (P’s > 0.9) scores and the dynamicity (“T”-index) of any connection. In contrast, *negative* associations were found between conscientiousness scores and the “T”-index across a series of networks including prefronto-parietal and prefronto-temporal networks, the default mode network, cingulate circuits, sensory-motor and limbic networks as well as posterior occipito-parietal circuits (P’s < 0.05, FDR) (Table 2, Fig. 4). Of note, we achieved good generalizability of our findings in all cases in which we found a significant effect in the GLM analyses (Table 2, Fig. 4) (median RRMSE = 0.14, maximum RRMSE = 0.20)^41^.

**Table 2:** Connections between pairs of large-scale networks ‘nodes’ (first and second column) in which negative associations between conscientiousness scores and the “T”-index of dynamicity were found while accounting for the other personality traits as well as variability in sex, age, handedness, years of education, and intelligence scores (FDR: false discovery rate correction for multiple comparisons across 105 connections).

ICA node#1	ICA node#2	P value (FDR)	Effect size	RRMSE
Extra-striate	Cingulate	0.012	0.154	0.167
Sensory/Motor-Limbic	Cingulate	0.012	0.157	0.188
Occipito-Frontal	Extra-striate	0.041	0.123	0.180
Occipito-Frontal	Fronto-Temporal	0.041	0.125	0.168
Default-Mode	Right-Fronto-Parietal	0.041	0.125	0.186
Default-Mode	Sensory/Motor-Limbic	0.041	0.122	0.185
Extra-striate	Anterior Frontal	0.041	0.131	0.154
Hippocampal-Cerebellar	Cingulate	0.041	0.130	0.189
Sensory-Motor	Sensory/Motor-Limbic	0.041	0.127	0.176
Occipito-Frontal	Striate	0.046	0.114	0.177
Occipito-Frontal	Sensory-Motor	0.046	0.118	0.145
Extra-striate	Occipito-Parietal	0.046	0.119	0.173
Extra-striate	Hippocampal-Cerebellar	0.046	0.114	0.175
Occipito-Parietal	Right-Fronto-Parietal	0.046	0.116	0.195
Occipito-Parietal	Sensory-Motor	0.046	0.114	0.149
Default-Mode	Striate	0.046	0.113	0.142
Fronto-Temporal	Sensory/Motor-Limbic	0.049	0.111	0.189

Effect sizes were estimated using partial correlations between the “T”-index and all independent variables in the model, ensuring full overlap between the estimated model coefficients and corresponding normalized effect sizes. Relative root mean square error (RRMSE) resulting from employing the estimated models on the test set is also reported. Model accuracy is considered excellent when RRMSE < 0.1, good when 0.1 < RRMSE < 0.2, fair when 0.2 < RRMSE < 0.3, and poor if RRMSE > 0.3^41^.

To exclude that our main findings were not confounded by personality-driven differences in head movement during scanning, we also tested for associations between the average root-mean-square (RMS) displacement across runs and conscientiousness scores. No significant correlation was found between RMS and conscientiousness (Pearson’s R = 0.04, P = 0.24).

## Discussion

We provide new evidence that people scoring high in conscientiousness display more stationary connectivity patterns across large-scale networks that have been implicated in cognitive control (i.e., prefronto-parietal, prefronto-temporal, default mode, anterior frontal, and cingulate networks), visuo-spatial and visuo-motor skills (striate, extra-striate, occipito-parietal, and occipito-frontal networks) and sensory/motor and emotional functions (sensory-motor and limbic networks). The relationship between conscientiousness and stationary connections was obtained using multi-variate statistical models that controlled for the remaining FFM traits (i.e., neuroticism, extraversion, openness, and agreeableness) and for variability in potentially confounding factors such as age, sex, handedness, education, and intelligence. Of note, the generalizability of our findings was supported by a ‘training’ and ‘test’ split validation approach.

We also found that, independently of personality differences, the connections across networks ‘feeding’ external inputs into the brain (occipito-parietal, occipito-frontal, striate, and extra-striate) and those regulating the motor output (i.e., sensory/motor-limbic-sensory/motor) displayed the highest levels of dynamicity. In contrast, the connections across networks involved in cognitive control (e.g., cingulate network, default mode network, anterior frontal circuits) showed more stationary connectivity patterns.

These data suggest that temporally resolved ‘connectomic’ indices are reliable markers that can be used as indicators of the cognitive and behavioural style across different people; for example, the ability of conscientious persons to efficiently pursue specific goals and maintain them consistent over time^42–45^. Furthermore, the current results support and extend our recent data showing that people scoring high in conscientiousness display increased nodal strength, local clustering, and betweenness-centrality across similar circuits^25^. Together, these findings mechanistically describe why conscientious people display a cognitive style that is resistant to the disruptive interference of incoming stimuli, which are often emotional in nature^46^.

This interpretation is in keeping with a theoretical framework positing that conscientiousness would contribute to the behavioural construct of ‘stability’, a meta-trait that would have evolved from the necessity to prevent the disruption of goals by interfering stimuli^46^. However, no significant results were obtained for the other ‘stability’ traits (i.e., low neuroticism and high agreeableness) which indicates that such effects do not necessarily extend to the meta-trait of ‘stability’. On the other hand, the temporal stationarity of the connectivity patterns in conscientious people can be interpreted in the context of previous studies showing that, across different samples and age-groups, conscientious people tend to display more ‘constant’ behavioural traits over time^47–51^.

The association between conscientiousness and stationary connectivity patterns was localized across networks that have been implicated in ‘top-down’ cognitive control, ‘bottom-up’ input-processing functions, and action generation. In particular, the fact that stationary interactions were found between prefronto-parietal and occipito-parietal/extra-striate circuits as well as between the default mode network and the sensory-motor, prefronto-parietal, and the visual striate networks is consistent with the notion that conscientious people maintain well the focus on non-immediate goals^1,52,53^.

At the same time, the stationary patterns of functional interactions between the cingulate cortices and sensory-motor limbic networks may help explaining why conscientious people successfully adjust their behavioural responses to different environmental contexts; for example, by delaying immediate gratification^1,52,53^. Stationarity in the patterns of ‘communication’ between memory circuits (i.e., hippocampal/cingulate networks) can also mediate a good performance during prospective memory tasks that require retrieval of complex sequences of planned actions^54–56^.

### Strengths and limitations

The use of a large sample of participants (*n* = 818), state-of art analytical pipelines (i.e., DCC model), and the inclusion of a training/test-set for assessing the generalizability of our findings are the main methodological advantages of this study.

The fact that conscientiousness was the sole personality trait related to time-variant connectivity measures is consistent with our recent data showing that conscientiousness was the only FFM factor linked to heightened static ‘connectomic’ metrics such as nodal strength, local clustering, and betweenness-centrality^25^. Nevertheless, our earlier and current studies *do not* necessarily imply that other FFM traits *do not* have any brain functional correlate as there can be several reasons for null results including: (i) type II errors; (ii) non-linear relationships between FFM traits and time-variant metrics, and (iii) the fact that correlations between time-variant functional measures and personality traits *might exist* but could only be revealed by more narrow measures of personality (i.e., facets within each of the five factors) or other (e.g., task-related) functional measures.

Another issue regards the level of anatomical ‘granularity’ of the brain networks that is required to reveal the relationship between the time-resolved patterns of connectivity and personality differences. In other words, one could hypothesize that it is: 1) the interaction between large-scale brain networks that relates to the dynamicity or stability of functional connections, and/or 2) the more fine-grained interplay between individual areas within a network (e.g., anterior and posterior components of the default mode network) that is linked to the cognitive/behavioural differences described by the FFM of personality. Although either possibilities are equally plausible, non-mutually exclusive, and potentially interesting to study, we assumed here that a large-scale network approach would have retained a sufficient level of anatomical detail to reveal the relationship between time-variant connectivity and differences in the FFM traits. The use of large-scale circuits (n = 15 ICA-derived networks) also enabled us to compare the findings from this study to those reported in the previous one^25^.

Finally, in this study, as in many others using rs-fMRI, people’s performance during fixation (i.e., eye tracking data) was not monitored. However, eye tracking data in a limited group of participants (*n* = 132), for which the 3 Tesla MRI data were also available, was subsequently collected during MRI scanning at ultra-high field (7 Tesla). Hence, we assessed the potential impact of eye movements during scanning on our main outcome measure (T-index) and found that it was negligible (see supplementary material). Although this result does not exclude *a priori* whether participants’ ability to fixate affected our current findings (as the session with the eye tracking data at 7 T and the sessions at 3 T were on separate days); it showed in principle that the calculation of the T-index is not influenced by eye movements during rs-fMRI.

The potential confound of eye movements or closure during scanning can also be partially mitigated via reliable procedures that correct for head movements that are typically associated with fatigue or lapses in concentration during scanning. In our study, the noise related to head movements was removed via the HCP-specific ICA-FIX automated algorithm (which has ~99% sensitivity and specificity in de-noising HCP data^57–59^). Furthermore, we did not find any relationship between conscientiousness and head displacement during scanning which suggests negligible effects of personality differences on mediating compliance during rs-fMRI scanning. However, as before, we cannot completely exclude *a priori* any interaction between the de-noising procedures and the correct preservation of the personality specific characteristics in the rs-fMRI signal.

## Conclusions

We found a negative association between conscientiousness scores and the dynamicity of the time-resolved functional connectivity patterns. Together with our recent results showing enhanced connectivity strength, local clustering, and betweenness centrality in cognitive networks in relation to conscientiousness, the current findings provide new mechanistic insights for the empirical observation that conscientious people are superb in maintaining long-term plans consistent over time.

## Methods

### Personality assessment

The FFM personality traits were assessed with the NEO five-factors inventory (NEO-FFI), which is composed by 60 items, 12 per each of the five factors^33,60^. For each item, participants reported their level of agreement on a 5-points Likert scale, from strongly disagree to strongly agree. This NEO instrument has been previously validated in the US and several other countries^35^. The recently discovered bug in the scoring of HCP agreeableness data was corrected prior to any further processing (personal communication on HCP mailing list on 03/09/2018 20:48 CEST). All data used in the present study are available for download from the Human Connectome Project (www.humanconnectome.org). Users must agree to data use terms for the HCP before being allowed access to the data and ConnectomeDB, details are provided at (https://www.humanconnectome.org/study/hcp-young-adult/data-use-terms). The HCP has implemented a two-tiered plan for data sharing, with different provisions for handling Open Access data and Restricted data (e.g., data related to family structure, age by year, handedness, etc). This study was carried out in compliance with the HCP restricted use data terms (https://www.humanconnectome.org/study/hcp-young-adult/document/wu-minn-hcp-consortium-restricted-data-use-terms). In particular, with reference to point 6 in the aforementioned document, local regulations at one of the PIs’ site (University of Rome Tor Vergata) do not require separate or individual ethics committee submission and/or approval.

### MRI scanning protocol and pre-processing

Rs-fMRI data were acquired using a 3T scanner (Siemens AG, Erlangen, Germany)^61^. Four runs of approximately 14 minutes and 24 seconds each were obtained. Subjects lied within the scanner with open eyes while fixating a bright central cross projected on a dark background. Oblique axial acquisitions were alternated between phase encoding in a right-to-left direction in one run and phase encoding in a left-to-right direction in the other run. Gradient-echo echo-planar imaging used the following parameters: TR = 720 ms, TE = 33.1 ms, flip angle = 52°, FOV = 208 × 180 mm, Matrix 104 × 90, Slice thickness = 2.0 mm; 72 slices; 2.0 mm isotropic voxels, Multiband factor = 8, Echo spacing = 0.58 ms, BW = 2290 Hz/Px. This resulted in 4,800 rs-fMRI volumes in total per subject, subdivided in 4 runs of 1,200 volumes each. Structural (T1-weighted) images and field maps were also acquired to aid data pre-processing. Within the HCP consortium, each 1,200 brain volumes run of each subject’s rsfMRI data was minimally pre-processed according to the latest version (3.1) of the HCP pipeline^62^.

Each dataset was temporally de-meaned and had variance normalization applied according to Beckmann and colleagues {Beckmann, 2004 #280}. Group-principal component analysis (PCA) output was generated by MIGP (MELODIC’s Incremental Group-PCA) from n = 818 participants. This comprises the top 4,500 weighted spatial eigenvectors from a group-averaged PCA^63^. The MIGP output was then fed into group-independent component analysis (ICA) using FSL’s MELODIC tool^64^, applying spatial-ICA at dimensionality of 15. Successively, the ICA maps (dimensionality: 15) were dual-regressed into each subject’s 4D dataset to give a set of 15 time-courses of 4,800 time points per subject. Further details regarding data acquisition and pre-processing can be found in the HCP S900 Release reference manual available at https://www.humanconnectome.org/. Node maps (Fig. 1) as well as node- and subject-specific time-series were obtained from the HCP database for further processing. From the same database we also obtained connectivity matrices computed through partial correlation (‘netmats2’, https://db.humanconnectome.org/data/projects/HCP_1200) to be included in our analysis.

### Estimation of time-variant functional connectivity

#### Dynamic connectivity estimation

To estimate DCC, we follow the methods outlined and the code distributed with the original publication^32^. GARCH volatility models assume that the conditional variance at time *t* is a linear combination of the past values of the conditional variance and of the past values of the squared process itself. The minimal univariate GARCH model takes the form:

1 $${y}_{t}={\sigma }_{t}{\varepsilon }_{t}$$

where ${\varepsilon }_{t}$ is a normal variable and the conditional variance ${\sigma }_{t}$ is a function of previous time-step of the signal and of the variance itself:

2 $${{\sigma }^{2}}_{t}=\omega +\alpha {{y}^{2}}_{t-1}+\beta {{\sigma }^{2}}_{t-1}$$

where *ω* > 0, *α* ≥ 0, *β* ≥ 0 and *α* + *β* < 1. The algorithm employed in this paper for any two fMRI time-series (i.e., the DCC algorithm)^65^ consists of the following three steps: i) given that GARCH models account for volatility around a mean, all signals are de-trended using an autoregressive integrated moving average (ARIMA) model; ii) univariate GARCH models are fitted to each of the two time-series and standardized residuals are computed, and iii) an exponentially weighted moving average (EWMA)-type method is applied to the standardized residuals to compute a non-normalized version of the time-varying correlation matrix ${{\bf{R}}}_{t}$,which is rescaled—see equations 19–24 in Lindquist *et al*.^32^—to obtain a conditional covariance. This procedure allows the estimation of time-varying, dynamical conditional covariance between the two components of ${y}_{t}$ which, in the case of two rs-fMRI time-series, can be employed as an estimate of dynamical connectivity, i.e. the DCC^32^ from the off-diagonal elements of the conditional covariance matric. The DCC which is an estimate of the association between the two time-series (i.e. connectivity) at the same temporal resolution as the original signals. All parameters are estimated using maximum likelihood methods.

After DCC estimation, for each DCC time-series (i.e., for each time-varying connection) we computed a non-stationarity index, term “T”-index” as explained in the following paragraph.

### T-index for quantifying non-stationarity

**Figure 5:**
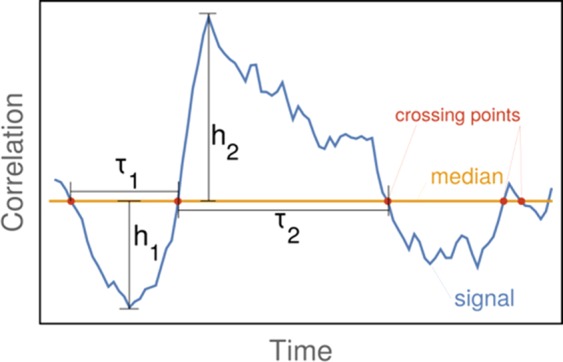
Example of excursions around the median value of a DCC estimate. Excursion length τ_1_ and τ_2_ and excursion magnitudes h_1_ and h_2_ are shown.

The non-stationarity T-index used here to evaluate the non-stationarity of time-varying connectivity was inspired by the T-index originally developed by Zalesky *et al*.^27^. Such T-index is based on the rationale that the non-stationarity property of a time-resolved correlation depends on large and prolonged excursions from the median connectivity value over-time. The larger and/or more prolonged such excursions in connectivity are, the more “dynamical” (i.e., non-stationary) the connection is considered to be. The T-index is defined and calculated as follows: (see also supplementary information in Zalesky *et al*.)^27^: (a) an excursion is defined by two consecutive crossing points around the median value (see Fig. 5); (b) the excursion’s length *τ* is defined by the time difference between two consecutive crossing points; (c) the excursion’s magnitude *h* is defined as the absolute difference between the highest or lowest time series correlation and its median value, within the context of each specific excursion. Figure 5 graphically summarizes these definitions.

Formally, the T-index of dynamicity is defined as follow:

3 $${\rm{T}} \mbox{-} {\rm{index}}=\frac{1}{N}{\sum }_{i}^{N}\,{h}_{i}\,{\tau }_{i}.$$

where N is the number of excursions.

To exclude potential confounding effects driven by differences in scan sessions (1st and 2nd half of the time-series), we conducted a series of additional analyses reported in supplementary material.

Moreover, to assess the impact of eye movements during scanning on the quantification of the T-index, we performed further analyses in a sub-group of people (*n* = 132) for which eye-tracking data were available during subsequent scanning at ultra-high-field (7 Tesla) (see supplementary material). Eye-tracking data (along with all other HCP data) are available as part of the 7 T data release at db.humanconnectome.org.

### Statistical analyses

#### Generation of train-test samples

To test the associations between non-stationarity measures and personality traits, as well as to evaluate the generalizability of our findings, the initial sample of *n* = 818 participants was split into two matched sub-samples: a ‘training’ set (75% of participants, *n* = 613) and a ‘test’ set (25% of participants, *n* = 205). The matched train/test split was generated by random sampling (with replacement) and subsequent comparison between the resulting train and test sets through nonparametric Mann-Whitney-U tests until no significant differences in the median “T”-index (across subjects) for any 105 possible connections was found. This ensured that the train and test set had comparable dependent variable distributions across all possible connections.

#### Inference of associations between non-stationarity and personality

The ‘training’ sample was used to estimate the association between the “T”-index (i.e., non-stationarity measure) described above and the FFM personality traits. Specifically, general linear models (GLMs), including each of the FFM traits as well as age, sex, handedness, education, and intelligence scores as covariates of no interest, were fitted to each connection (dependent variable: non-stationarity index “T”). The resulting P-values were corrected for multiple comparisons across all possible 105 connections using a false discovery rate (FDR) procedure. Associations surviving a threshold of P < 0.05 (FDR-corrected) were considered statistically significant. The results were a set of FDR corrected P-values as well as multivariate regression coefficients for each connection. For each regression (i.e., for each connection), effect sizes were estimated using the absolute values of partial correlations between the “T”-index and all independent variables in the model. As partial correlation is defined through linear regression, this ensured full overlap between the estimated model coefficients and corresponding normalized effect sizes.

#### Generalizability to unseen test set

**Figure 6:**
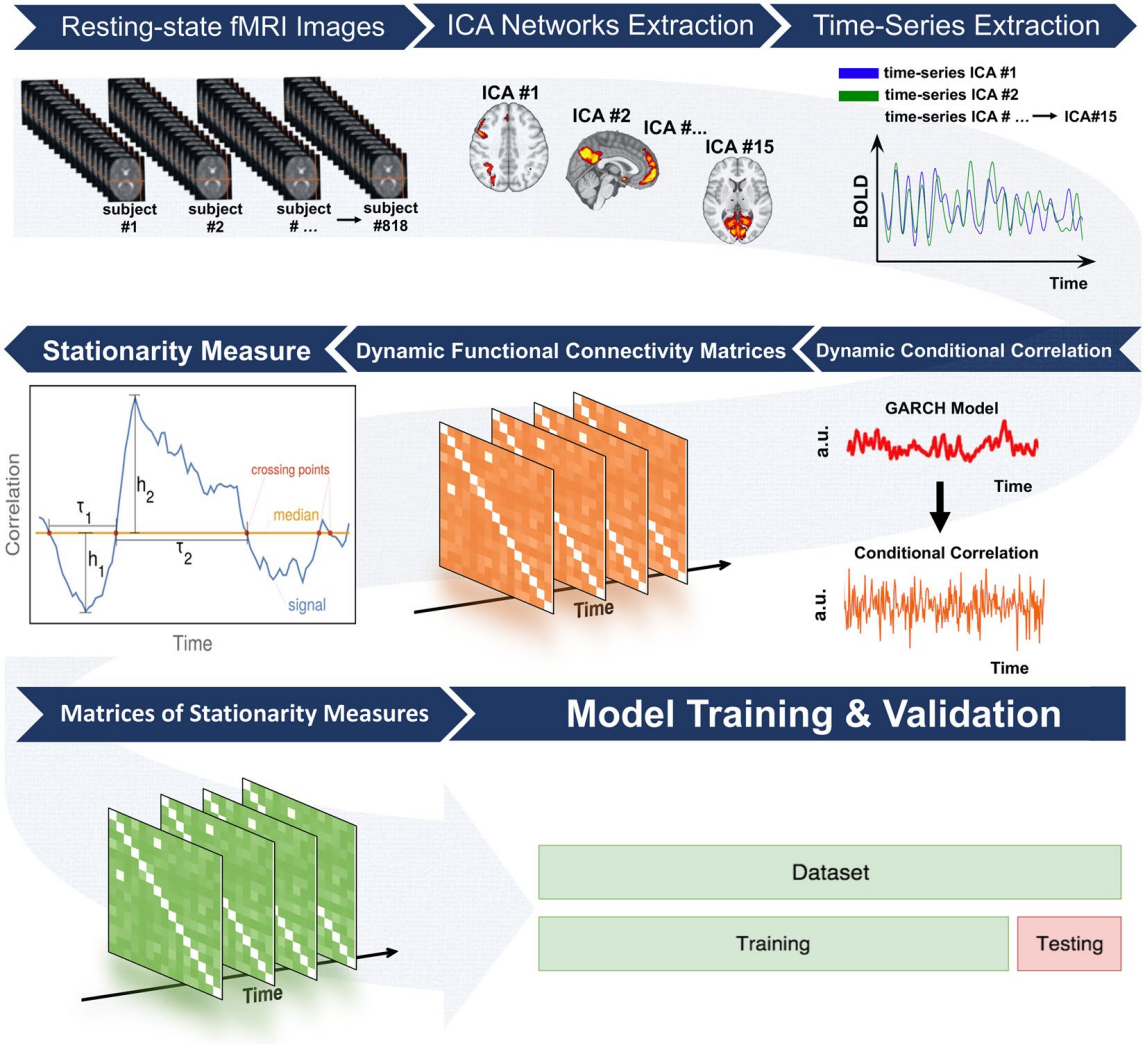
Image analysis workflow. The resting-state functional magnetic imaging (fMRI) data were initially pre-processed and next a set of 15 separate brain circuits were extracted via independent components analysis (ICA). Next, time-series from each ICA brain circuit were obtained from each individual and fed into dynamic conditional correlation (DCC) models. This led to 15 × 15 time-variant functional connectivity matrices at the single-subject level that were then used to estimate non-stationarity “T”-index as in Zalesky *et al*.^27^. Finally, these “T”-index values were analyzed in conjunction with the traits of the five-factor-model of personality at the group level. The generalization ability of the model was evaluated using a using a train/test split approach (see the statistical analyses section of the methods for further information).

Finally, the ‘test’ sample (i.e., in an unseen group of subjects to which the model was completely ‘agnostic’) was employed to assess the generalizability of the multivariate models fitted on the ‘training’ set. To this end, the GLMs fitted in the training set were used to estimate the “T”-indices in the ‘test’ sample using the demographic variables and personality scores of the ‘test’ sample as inputs (i.e., the rs-fMRI data of the ‘train’ sample was not employed in this procedure). The similarity between ‘real’ “T”-indices (i.e., computed using rs-fMRI data from the ‘test’ sample) and ‘estimated’ time-variant indices (i.e., predicted using the GLM coefficients fitted on ‘training’ data only) was assessed through the relative root mean square error (RRMSE), a normalized version of the root mean squared error which is often used as a measure of the differences between predicted and observed values. Model accuracy can be considered excellent when RRMSE < 0.1, good when 0.1 < RRMSE < 0.2, fair when 0.2 < RRMSE < 0.3, and poor if RRMSE > 0.3^41^. This ensured comparability of generalization capability across the personality traits. The image analysis pipeline is summarized in Fig. 6.

## Supplementary information

Supplementary Infomation
