# Supplementary material for: Time-resolved connectome of the five-factor model of personality
Source: Sci Rep. 2019 Oct 21;9:15066. doi: 10.1038/s41598-019-51469-2 (PMC6803687; doi:10.1038/s41598-019-51469-2)

**Time-resolved connectome of the five-factor model of personality**

L. Passamonti^1,2^, R. Riccelli^3^, I. Indovina^3,4^, A. Duggento^5^, A. Terracciano^6^, and N. Toschi ^5,7^

**Authors’ addresses:**

^1^Institute of Bioimaging & Molecular Physiology, National Research Council, Milano, Italy

^2^Department of Clinical Neurosciences, University of Cambridge, Cambridge, UK

^3^Laboratory of Neuromotor Physiology, IRCCS Santa Lucia Foundation, 00179, Rome, Italy

^4^Saint Camillus International University of Health Sciences, 00131, Rome, Italy

^5^Department of Biomedicine & Prevention, University "Tor Vergata", Rome, Italy

^6^Department of Geriatrics, Florida State University College of Medicine, Tallahassee, USA

^7^Department of Radiology, Martinos Center for Biomedical Imaging, Boston & Harvard medical School, Boston, USA

**Corresponding Author:**

Dr. Luca Passamonti

Department of Clinical Neurosciences

University of Cambridge

Cambridge, CB2 0SZ, UK

Telephone: +44.01223.330293

Email: [lp337@medschl.cam.ac.uk](mailto:lp337@medschl.cam.ac.uk)

**Running title:** Time-variant connectivity and the Big-Five

**Subject:** Biological sciences/Neurosciences/Network models/Personality Neuroscience

**Techniques:** Physical Sciences techniques [statistical techniques]

Life sciences techniques, Medical imaging [Magnetic resonance imaging]

**Total word count:** (excluding abstract, figures, table, and references): 4,324

**Abstract word count:** 154

**Number of pages:** 27

**Figures:** 6, **Tables**: 2, **Supplementary Material**: 1 supp text, 2 supp figures, 2 supp tables

**Supplementary Table 1.** Anatomical Localization of topologically connected clusters in each of the 15 Independent Component Analysis (ICA) components after thresholding at the 99th percentile. MAX: maximum value of dual regression coefficient within specific cluster. MNI: Montreal Neurological Institute. All coordinates are in mm. L, left hemisphere, R, right hemisphere.

| **ICA #1**  **Occipito-Frontal Circuit** | N° of  voxels | MAX | MNI  X | MNI  Y | MNI  Z |
| --- | --- | --- | --- | --- | --- |
| Visual cortex V2 BA18 R | 1326 | 62.2 | 14 | -92 | 30 |
| Caudate R | 291 | 63.7 | 10 | 0 | 12 |
| Visual cortex V4 R | 239 | 42.4 | 24 | -74 | -16 |
| Visual cortex V3V L | 150 | 39.9 | -20 | -70 | -14 |
| Amygdala superficial group L | 147 | 56.2 | -24 | 0 | -10 |
| Caudate L | 145 | 62.4 | -8 | 2 | 8 |
| Amygdala superficial group R | 67 | 57.3 | 24 | 2 | -10 |
| Para-Cingulate Gyrus | 55 | 29.6 | 0 | 32 | 32 |
| Inferior Frontal Gyrus BA44 R | 39 | 30.7 | 52 | 10 | 26 |
| Brain stem | 36 | 40.4 | 6 | -10 | -16 |
| Inferior Frontal Gyrus BA45 L | 34 | 34.9 | -48 | 16 | -6 |
| Cerebellum L | 33 | 42.5 | -2 | -52 | -34 |
| Frontal operculum R | 21 | 35.5 | 50 | 18 | -6 |
| Putamen R | 20 | 28 | 22 | 6 | 6 |
| Frontal Operculum L | 15 | 33 | -32 | 20 | 10 |
| Premotor cortex BA6 R | 15 | 28.3 | 2 | 18 | 50 |
| Lateral geniculate body L | 14 | 35.1 | -22 | -26 | -8 |
| Inferior Frontal Gyrus BA44 L | 13 | 28.8 | -50 | 6 | 24 |
| Cerebellum R | 13 | 29.8 | 18 | -42 | -46 |
| Lateral Occipital Cortex L | 12 | 28.4 | -48 | -82 | 4 |

| **ICA #2**  **Default Mode Network** | N° of  voxels | MAX | MNI  X | MNI  Y | MNI  Z |
| --- | --- | --- | --- | --- | --- |
| Frontal Pole / medial prefrontal cortex | 1285 | 73.4 | 0 | 62 | -6 |
| Posterior Cingulate Gyrus L | 865 | 70.4 | -2 | -52 | 16 |
| Inferior parietal lobule L | 472 | 73.5 | -46 | -68 | 44 |
| Frontal Pole R | 48 | 49.7 | 18 | 42 | 50 |
| Inferior parietal lobule R | 47 | 46.2 | 54 | -64 | 36 |
| Subgenual Anterior Cingulate Cortex | 26 | 74.4 | 0 | 6 | -12 |
| Inferior frontal gyrus BA45 L | 18 | 44.7 | -40 | 30 | -14 |

| **ICA #3**  **Extra-striate Visual Circuit** | N° of  voxels | MAX | MNI  X | MNI  Y | MNI  Z |
| --- | --- | --- | --- | --- | --- |
| Occipital Gyrus L | 1424 | 65.7 | -26 | -86 | -20 |
| Occipital Gyrus R | 1382 | 65 | 26 | -94 | 0 |

| **ICA #4**  **Occipito-Parietal Circuit** | N° of voxels | MAX | MNI X | MNI Y | MNI Z |
| --- | --- | --- | --- | --- | --- |
| Inferior parietal lobule R | 1073 | 57.8 | 34 | -86 | 28 |
| Visual cortex V2 BA18 L | 772 | 57.1 | -26 | -88 | 26 |
| Visual cortex V2 BA18 R | 488 | 45 | 30 | -56 | -6 |
| Visual cortex V4 L | 457 | 39.9 | -28 | -64 | -6 |
| Premotor cortex R | 11 | 27.6 | 52 | 8 | 38 |

| **ICA #5**  **Left Fronto-Parietal Circuit** | N° of voxels | MAX | MNI X | MNI Y | MNI Z |
| --- | --- | --- | --- | --- | --- |
| Dorsolateral prefrontal cortex L | 1619 | 79.7 | -52 | 14 | 32 |
| Inferior parietal lobule L | 605 | 48.2 | -40 | -56 | 56 |
| Medial Superior Frontal Gyrus L | 172 | 55.5 | -2 | 26 | 46 |
| Inferior Frontal Gyrus BA45 R | 89 | 35.5 | 48 | 32 | 20 |
| Superior Frontal Gyrus L | 88 | 47.6 | -28 | 14 | 62 |
| Frontal Orbital Cortex L | 46 | 46 | -34 | 36 | -10 |
| Medial Frontal Pole R | 32 | 36.5 | 2 | 58 | 14 |
| Inferior Temporal Gyrus L | 30 | 34.2 | -54 | -44 | -12 |
| Middle Temporal Gyrus L | 23 | 29.5 | -62 | -54 | -8 |
| Inferior Frontal gyrus BA44 L | 18 | 30.1 | -38 | 22 | -2 |
| Cerebellum R | 16 | 33 | 10 | -78 | -30 |
| Inferior Temporal Gyrus L | 16 | 29.3 | -50 | -60 | -20 |
|  | | | | | |
| **ICA #6**  **Right Fronto-Parietal Circuit** | N° of  voxels | MAX | MNI  X | MNI  Y | MNI  Z |
| Frontal Pole R | 1939 | 63 | 42 | 56 | 0 |
| Inferior parietal lobule R | 701 | 47.7 | 42 | -62 | 52 |
| Cerebellum L | 30 | 32.6 | -8 | -78 | -30 |
| Inferior Frontal Gyrus BA45 R | 30 | 30.1 | 56 | 22 | 4 |
| Cerebellum L | 24 | 29.2 | -36 | -68 | -40 |
| Middle Temporal Gyrus R | 23 | 30.8 | 64 | -28 | -2 |
| Frontal Orbital Cortex L | 12 | 30.9 | -32 | 36 | -10 |
|  | | | | | |
| **ICA #7**  **“Salience” Network** | N° of  voxels | MAX | MNI  X | MNI  Y | MNI  Z |
| Supra-marginal Gyrus L | 535 | 50 | -60 | -36 | 32 |
| Insula L | 474 | 67.1 | -40 | -12 | -8 |
| Inferior parietal lobule R | 434 | 60.1 | 56 | -30 | 34 |
| Inferior Frontal Gyrus BA44 R | 418 | 74.2 | 58 | 12 | 0 |
| Inferior Frontal Gyrus BA45 L | 212 | 41 | -48 | 42 | 12 |
| Pre-motor cortex BA6 R | 191 | 38.8 | 2 | 12 | 42 |
| Frontal Pole R | 129 | 43.6 | 50 | 44 | 4 |
| Superior parietal lobule R | 101 | 48.1 | 16 | -34 | 42 |
| Insula R | 100 | 68.5 | 42 | -8 | -10 |
| Superior parietal lobule L | 81 | 51.6 | -14 | -30 | 38 |
| Orbitofrontal cortex L | 35 | 36.3 | -32 | 38 | -10 |
| Middle Temporal Gyrus L | 33 | 32.3 | -60 | -62 | 0 |
| Anterior Cingulate Cortex L | 18 | 30.8 | -10 | 8 | 38 |
| Pre-motor cortex BA6 L | 17 | 28.4 | -2 | 6 | 66 |
| Amygdala L | <15 | 26.8 | -20 | 0 | -14 |
| Amygdala R | <15 | 25.9 | 22 | 2 | -14 |
|  | | | | | |
| **ICA #8**  **Striate Visual Circuit** | N° of  voxels | MAX | MNI  X | MNI  Y | MNI  Z |
| Intra-Calcarine Cortex R / L | 2716 | 134 | 10 | -72 | 14 |
| Thalamus L | 20 | 60.8 | -12 | -32 | -4 |
| Thalamus L | 19 | 58 | -10 | -16 | 8 |
| Thalamus R | 17 | 60.4 | 14 | -30 | -4 |

| **ICA #9**  **Hippocampal-Cerebellar Circuit** | N° of  voxels | MAX | MNI  X | MNI  Y | MNI  Z |
| --- | --- | --- | --- | --- | --- |
| Cerebellum L | 1439 | 41.4 | -34 | -68 | -22 |
| Thalamus L | 267 | 49.7 | -10 | -4 | 14 |
| Hippocampus R | 249 | 31.9 | 26 | -20 | -16 |
| Hippocampus subiculum L | 221 | 34.8 | -14 | -38 | 2 |
| Insula L | 115 | 30.3 | -40 | -20 | -2 |
| Secondary somatosensory cortex / Parietal operculum R | 111 | 32.2 | 36 | -28 | 20 |
| Thalamus | 42 | 34.8 | 0 | -24 | -2 |
| Insula L | 33 | 24.8 | -30 | 22 | -6 |
| Cerebellum R | 32 | 35.7 | 30 | -40 | -40 |
| Insula R | 30 | 23 | 32 | 22 | -4 |
| Cerebellum L | 25 | 28.1 | -32 | -42 | -40 |
| Caudate L | 22 | 22.3 | -16 | 6 | 22 |
| Caudate R | 19 | 25.3 | 18 | 4 | 24 |
| Hippocampus subiculum R | 16 | 23.2 | 12 | -30 | -2 |
| Cerebellum R | 14 | 28.6 | 16 | -44 | -46 |
| Brain-Stem | 13 | 26.1 | 0 | -34 | -8 |
| Cerebellum L | 12 | 29.6 | -12 | -46 | -44 |
| Frontal Operculum R | 11 | 21.3 | 50 | 18 | -6 |
|  | | | | | |
| **ICA #10**  **Para-hippocampal Circuit** | N° of  voxels | MAX | MNI  X | MNI  Y | MNI  Z |
| Posterior Cingulate Gyrus R | 608 | 72.8 | 8 | -48 | 4 |
| Superior parietal lobule L | 510 | 42.4 | -6 | -66 | 62 |
| Precuneus L | 403 | 69.1 | -16 | -58 | 18 |
| Middle Frontal Gyrus R | 319 | 43.2 | 28 | 32 | 52 |
| Inferior parietal lobule L | 301 | 55.2 | -34 | -84 | 38 |
| Inferior parietal lobule R | 253 | 54 | 42 | -80 | 32 |
| Premotor cortex BA6 L | 127 | 35.6 | -22 | 10 | 58 |
| Parahippocampal Gyrus L | 113 | 54 | -32 | -40 | -12 |
| Parahippocampal Gyrus R | 92 | 47.5 | 34 | -36 | -14 |
| Hippocampus subiculum R | 17 | 37.2 | 22 | -20 | -16 |
| Cerebellum L | 16 | 42.5 | -10 | -48 | -44 |
| Posterior Cingulate Cortex | 13 | 32.7 | 0 | -24 | 28 |
| Cerebellum R | 12 | 35.3 | 12 | -46 | -44 |
|  | | | | | |
| **ICA #11**  **Sensory-Motor Circuit** | N° of voxels | MAX | MNI X | MNI Y | MNI Z |
| Primary somatosensory cortex BA2 L | 1353 | 46.3 | -52 | -30 | 52 |
| Primary somatosensory cortex BA2 R | 769 | 46.3 | 56 | -24 | 52 |
| Premotor cortex BA6 R | 343 | 36.4 | 40 | -22 | 66 |
| Premotor cortex BA6 L | 91 | 32.3 | -54 | 8 | 38 |
| Inferior Frontal Gyrus BA44 R | 37 | 25.6 | 54 | 10 | 24 |
| Secondary somatosensory cortex / Parietal operculum L | 36 | 24.7 | -50 | -20 | 20 |
| Medial Premotor cortex BA6 | 28 | 22.2 | 0 | -6 | 56 |
| Secondary somatosensory cortex / Parietal operculum L | 27 | 38.5 | -38 | -4 | 16 |
| Superior parietal lobule R | 24 | 22.3 | 18 | -70 | 54 |
| Secondary somatosensory cortex / Parietal operculum R | 20 | 33.3 | 40 | -2 | 16 |
| Secondary somatosensory cortex / Parietal operculum R | 15 | 21 | 52 | -16 | 18 |

| Superior parietal lobule L | 14 | 20.5 | -16 | -72 | 54 |
| --- | --- | --- | --- | --- | --- |
|  | | | | | |
| **ICA #12**  **Fronto-Temporal Circuit** | N° of voxels | MAX | MNI X | MNI Y | MNI Z |
| Broca's area BA45 L | 868 | 72.9 | -48 | 20 | -6 |
| Middle Temporal Gyrus L | 526 | 46 | -48 | -32 | -2 |
| Premotor cortex BA6 L | 468 | 44 | -4 | 24 | 64 |
| Middle Temporal Gyrus R | 392 | 50.1 | 48 | -22 | -8 |
| Broca's area BA45 R | 309 | 46.3 | 50 | 22 | -6 |
| Temporal Pole L | 85 | 32.9 | -48 | 2 | -24 |
| Premotor cortex BA6 L | 52 | 33.1 | -44 | 2 | 56 |
| Superior Frontal Gyrus L | 48 | 32.1 | -2 | 54 | 30 |
| Broca's area BA45 R | 33 | 31 | 54 | 22 | 28 |
| Inferior parietal lobule L | 15 | 29.5 | -42 | -54 | 22 |
|  | | | | | |
| **ICA #13**  **Sensory/Motor-Limbic Circuit** | N° of voxels | MAX | MNI X | MNI Y | MNI Z |
| Primary Somatosensory Cortex (BA3) | 1232 | 125 | 52 | -6 | 26 |
| Primary Somatosensory Cortex (BA3) | 1220 | 119 | -54 | -8 | 24 |
| Pre-motor Cortex (BA6) | 138 | 39.1 | 2 | 2 | 64 |
| Secondary Somatosensory Cortex /Parietal Operculum (OP2) | 81 | 55.6 | -34 | -30 | 18 |
| Amygdala, superficial group | 30 | 57.9 | 26 | 0 | -10 |
| Amygdala, superficial group | 26 | 50.7 | -26 | -2 | -10 |
| Caudate Nucleus | 25 | 51.5 | 10 | 0 | 12 |
| Caudate Nucleus | 24 | 41.7 | -8 | 2 | 8 |
| Primary Motor Cortex (BA4) | 19 | 32.5 | 20 | -28 | 58 |
| Primary Motor Cortex (BA4) | 15 | 41 | -18 | -30 | 60 |
|  | | | | | |
| **ICA #14**  **Anterior Frontal Circuit** | N° of voxels | MAX | MNI X | MNI Y | MNI Z |
| Middle Frontal Gyrus L | 1608 | 41 | -36 | 34 | 40 |
| Paracingulate Gyrus L | 425 | 39.2 | -2 | 28 | 42 |
| Frontal Pole R | 196 | 30.4 | 32 | 52 | 28 |
| Inferior parietal lobule L | 179 | 29.2 | -54 | -50 | 48 |
| Premotor cortex BA6 R | 85 | 27.7 | 18 | 12 | 68 |
| Inferior Frontal Gyrus BA45 R | 75 | 24.4 | 54 | 24 | 28 |
| Frontal Pole R | 49 | 24.5 | 2 | 60 | -6 |
| Inferior Frontal Gyrus BA45 L | 35 | 30.9 | -50 | 16 | -4 |
| Amygdala superficial group R | 25 | 31 | 16 | -4 | -14 |
| Medial Precentral Gyrus | 21 | 24.8 | 0 | -34 | 48 |
| Amygdala superficial group L | 14 | 26.8 | -16 | -2 | -16 |
| Frontal Pole L | 14 | 26.4 | -30 | 50 | -12 |
| Subgenual Anterior Cingulate Cortex | 11 | 28.7 | 0 | 6 | -12 |
|  | | | | | |
| **ICA #15**  **Cingulate Cortex Circuit** | N° of voxels | MAX | MNI X | MNI Y | MNI Z |
| Posterior Cingulate Gyrus | 2353 | 114 | 0 | -30 | 26 |
| Paracingulate Gyrus R | 138 | 32.4 | 2 | 24 | 36 |
| Posterior Cingulate Gyrus L | 110 | 38.5 | -14 | -46 | 34 |

| Frontal Operculum R | 56 | 37.8 | 58 | 12 | -2 |
| --- | --- | --- | --- | --- | --- |
| Anterior intra-parietal sulcus L | 34 | 27.6 | -34 | -58 | 40 |
| Frontal Operculum L | 26 | 32.1 | -58 | 8 | 0 |
| Medial Precentral Gyrus | 21 | 30.8 | 0 | -34 | 48 |
| Insula R | 12 | 28.8 | 42 | -8 | -10 |
| Paracingulate Gyrus L | 12 | 27.1 | -10 | 26 | 28 |

To explore potential confounds driven by differences in series length, we , we re-computed the T-index for all DCC series (15x14/2 = 105 series, i.e. one for each connection) for all subjects in the training sample, and then compared the T-index of the first vs. second half of the time-series (across subjects and for each connection) using the non-parametric Mann-Whitney test (with False Discovery Rate, FDR, correction for multiple comparisons).

We found only 5 (out of 105) differences in the T-indices between the first and second half of the time-series (see Supplementary Table 2 below).

**Supplementary Table 2.** Connections that showed statistically significant difference between the first and second session in terms of effect size % difference. ICA (independent component analysis) -node (set of brain areas identified via the ICA). FDR, false discovery rate.

| **FDR Corrected**  **P-values** | **ICA node#1** | **ICA node#2** | **Effect Size**  **(% difference between first and second session)** |
| --- | --- | --- | --- |
| 0.002 | 3 | 15 | 8% |
| 0.005 | 3 | 13 | 9% |
| 0.02 | 8 | 15 | -8% |
| 0.05 | 9 | 13 | 11% |
| 0.05 | 2 | 7 | -8% |

The histogram of the (%) effect size between the first and second session (for all connections) is also reported in Supplementary Figure 1.

**Supplementary Figure 1**. Histogram representing all the connections in relation to the effect size (% difference between the T index calculated using the first and second half of the data). The connections that showed a statistically significant difference between the first and second half of the data are listed in Supplementary Table 1.


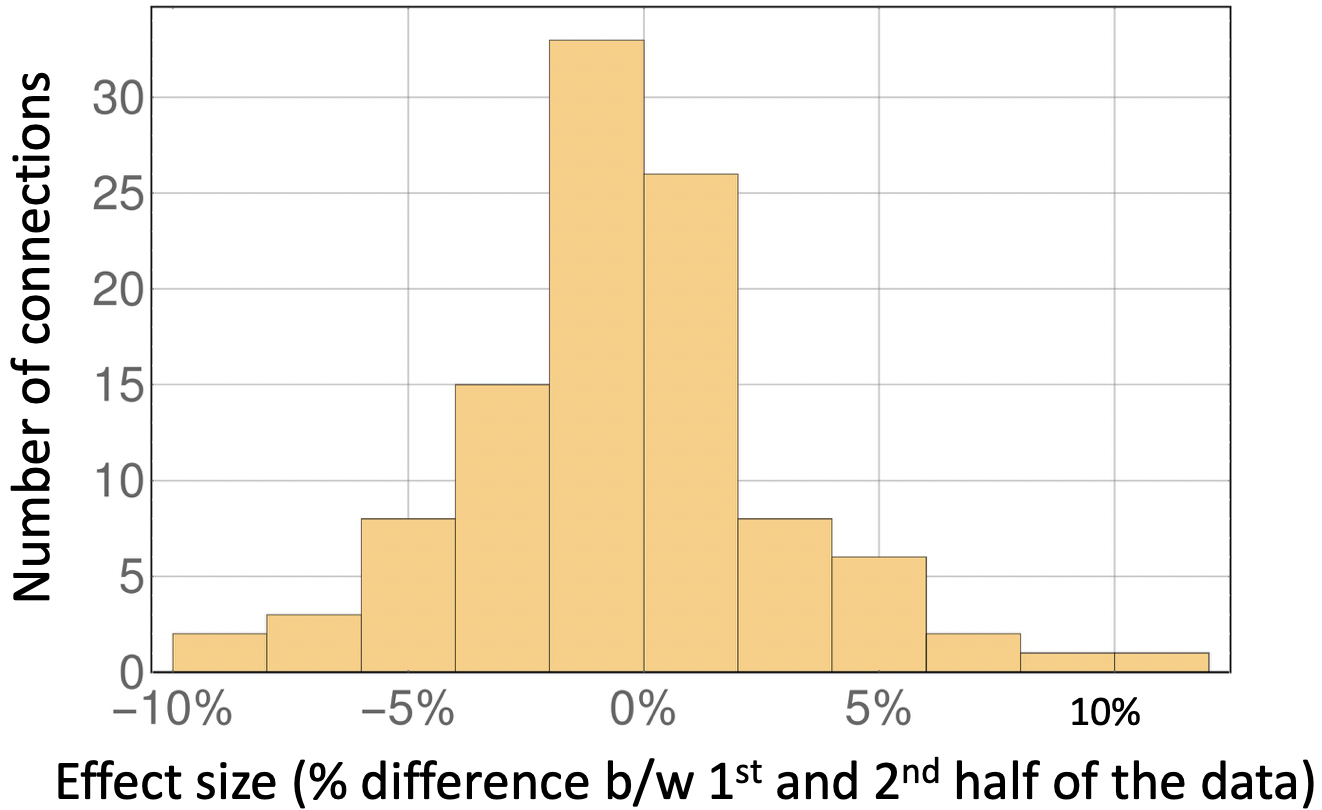


Considering that the main effects size reported in Table 2 of the main manuscript ranged from 0.11 to 0.15, we believe that the impact of the session on our main results was minimal, in terms of absolute effect sizes (8-11%) as well as the extent of connections involved.

Next, we assessed, in the subset of individuals who had eye tracking data at 7T (*n=132*), how eye movements during scanning might have potentially influenced the calculation of the T-index.

To this end, for each subject and run where eye-tracking data were available, we: 1) smoothed the high-frequency recording of {x,y} coordinate time series of pupil positions with a moving average filter and down-sampled the data at 0.72s (to avoid the introduction of possible bias due to different sampling between the eye-tracking and fMRI data) 2) calculated the absolute displacement as *displacement* = Sqrt[x^2 + y^2] , 3) computed the T-index for the *displacement* series and averaged it across runs for each subject, 4) calculated the correlation between this subject-wise T-index (from eye-tracking data) and the subject-wise T-index for all DCC series (15x14/2 = 105 series, i.e. one for each connection) for the sample in which eye-tracking data were available (n=132).

In summary, no significant correlations between subject-wises T-indices (from eye-tracking data) and subject-wise T-indices for all DCC series were found (lowest P=0.14, highest Pearson’s r=0.22) (see Supplementary Table 2). This suggests that the impact of eye movements during scanning on the T-index calculation tends to be minimal or negligible.

**Supplementary Table 3.** Correlations coefficients and p-values for the correlations between the subject-wise T-index (from eye-tracking data) and the subject-wise T-index for all DCC series per each pair of connections.

| **P-value (FDR Corrected)** | **Correlation** | **ICA1** | **ICA2** |
| --- | --- | --- | --- |
| 0.148 | 0.22 | 3 | 8 |
| 0.298 | 0.10 | 1 | 10 |
| 0.298 | 0.20 | 7 | 9 |
| 0.298 | 0.21 | 1 | 7 |
| 0.298 | 0.21 | 7 | 11 |
| 0.298 | 0.22 | 5 | 13 |
| 0.298 | 0.22 | 6 | 7 |
| 0.358 | 0.12 | 9 | 11 |
| 0.358 | 0.14 | 6 | 11 |
| 0.358 | 0.15 | 3 | 9 |
| 0.358 | 0.15 | 4 | 7 |
| 0.358 | 0.17 | 4 | 9 |
| 0.358 | 0.19 | 5 | 14 |
| 0.451 | 0.13 | 10 | 13 |
| 0.463 | 0.08 | 2 | 8 |
| 0.466 | 0.16 | 7 | 13 |
| 0.470 | 0.02 | 8 | 9 |
| 0.470 | 0.09 | 6 | 8 |
| 0.470 | 0.11 | 10 | 12 |
| 0.470 | 0.13 | 1 | 11 |
| 0.470 | 0.14 | 8 | 11 |
| 0.470 | 0.14 | 9 | 10 |
| 0.470 | 0.14 | 3 | 14 |
| 0.470 | 0.15 | 5 | 9 |
| 0.470 | 0.17 | 12 | 13 |
| 0.470 | 0.22 | 1 | 6 |
| 0.479 | -0.14 | 1 | 15 |
| 0.479 | -0.11 | 5 | 15 |
| 0.479 | 0.04 | 2 | 13 |
| 0.479 | 0.06 | 1 | 2 |
| 0.479 | 0.07 | 3 | 4 |
| 0.479 | 0.08 | 1 | 5 |
| 0.479 | 0.10 | 8 | 10 |
| 0.479 | 0.11 | 7 | 8 |
| 0.479 | 0.14 | 4 | 10 |
| 0.479 | 0.14 | 3 | 7 |
| 0.479 | 0.18 | 6 | 9 |
| 0.479 | 0.20 | 3 | 10 |
| 0.479 | 0.22 | 1 | 9 |
| 0.489 | -0.10 | 14 | 15 |
| 0.495 | 0.09 | 7 | 12 |
| 0.499 | -0.14 | 3 | 6 |
| 0.499 | 0.06 | 2 | 4 |
| 0.499 | 0.11 | 3 | 12 |
| 0.540 | -0.05 | 5 | 7 |
| 0.555 | 0.08 | 2 | 11 |
| 0.577 | -0.11 | 7 | 10 |
| 0.577 | 0.10 | 11 | 14 |
| 0.577 | 0.16 | 8 | 14 |
| 0.600 | 0.05 | 1 | 12 |
| 0.631 | 0.05 | 11 | 12 |
| 0.671 | -0.17 | 6 | 10 |
| 0.676 | 0.06 | 13 | 15 |
| 0.676 | 0.19 | 6 | 13 |
| 0.695 | 0.09 | 1 | 14 |
| 0.704 | 0.04 | 4 | 14 |
| 0.709 | 0.19 | 11 | 15 |
| 0.716 | 0.02 | 11 | 13 |
| 0.737 | 0.02 | 6 | 12 |
| 0.737 | 0.04 | 5 | 12 |
| 0.748 | 0.10 | 10 | 14 |
| 0.762 | 0.14 | 2 | 7 |
| 0.764 | -0.09 | 2 | 10 |
| 0.764 | 0.00 | 4 | 13 |
| 0.764 | 0.02 | 4 | 6 |
| 0.764 | 0.06 | 7 | 15 |
| 0.764 | 0.07 | 9 | 15 |
| 0.764 | 0.17 | 3 | 15 |
| 0.798 | -0.10 | 8 | 15 |
| 0.798 | -0.07 | 9 | 14 |
| 0.798 | -0.04 | 4 | 5 |
| 0.798 | -0.02 | 1 | 8 |
| 0.798 | -0.01 | 9 | 12 |
| 0.798 | 0.03 | 10 | 15 |
| 0.798 | 0.04 | 5 | 8 |
| 0.798 | 0.08 | 1 | 13 |
| 0.798 | 0.12 | 9 | 13 |
| 0.820 | -0.04 | 4 | 12 |
| 0.822 | -0.05 | 6 | 15 |
| 0.822 | -0.03 | 1 | 3 |
| 0.822 | -0.02 | 13 | 14 |
| 0.822 | 0.01 | 8 | 13 |
| 0.822 | 0.01 | 5 | 6 |
| 0.822 | 0.02 | 10 | 11 |
| 0.822 | 0.02 | 4 | 11 |
| 0.826 | -0.03 | 4 | 15 |
| 0.841 | -0.07 | 2 | 9 |
| 0.880 | -0.05 | 12 | 15 |
| 0.901 | -0.02 | 2 | 15 |
| 0.907 | 0.05 | 5 | 11 |
| 0.928 | 0.07 | 2 | 14 |
| 0.931 | -0.04 | 3 | 13 |
| 0.937 | 0.06 | 2 | 6 |
| 0.957 | -0.10 | 4 | 8 |
| 0.957 | 0.02 | 5 | 10 |
| 0.974 | -0.08 | 1 | 4 |
| 0.974 | -0.05 | 12 | 14 |
| 0.974 | -0.03 | 8 | 12 |
| 0.974 | -0.03 | 3 | 11 |
| 0.974 | 0.03 | 6 | 14 |
| 0.974 | 0.06 | 2 | 3 |
| 0.974 | 0.07 | 3 | 5 |
| 0.977 | 0.02 | 2 | 5 |
| 0.989 | 0.01 | 2 | 12 |
| 0.989 | 0.02 | 7 | 14 |

The histogram for the analyses exploring the relationship between subject-wise T-index (from eye-tracking data) and the subject-wise T-index for all DCC series is reported in Supplementary Figure 2.

**Supplementary Figure 2**. Histogram representing correlation coefficients and p-values between the subject-wise T-index (from eye-tracking data) and the subject-wise T-index for all DCC series per each pair of connections.


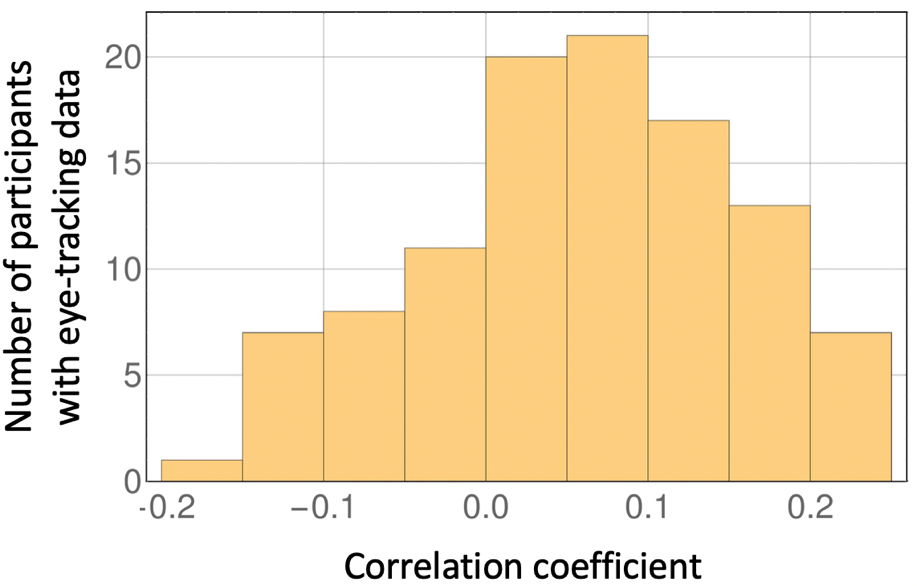

Supplement: Supplementary file 1 — Supplementary Infomation [file 41598_2019_51469_MOESM1_ESM.docx]
